# Supplementary material for: Vapor-deposited zeolitic imidazolate frameworks as gap-filling ultra-low-k dielectrics
Source: Nat Commun. 2019 Aug 19;10:3729. doi: 10.1038/s41467-019-11703-x (PMC6700180; doi:10.1038/s41467-019-11703-x)
Supplement: Supplementary file 1 — Supplementary Information [file 41467_2019_11703_MOESM1_ESM.pdf]

Electronic Supplementary Information for

Vapor-deposited zeolitic imidazolate frameworks as gap-filling ultra-low-k dielectrics

Mikhail Krishtab et al.

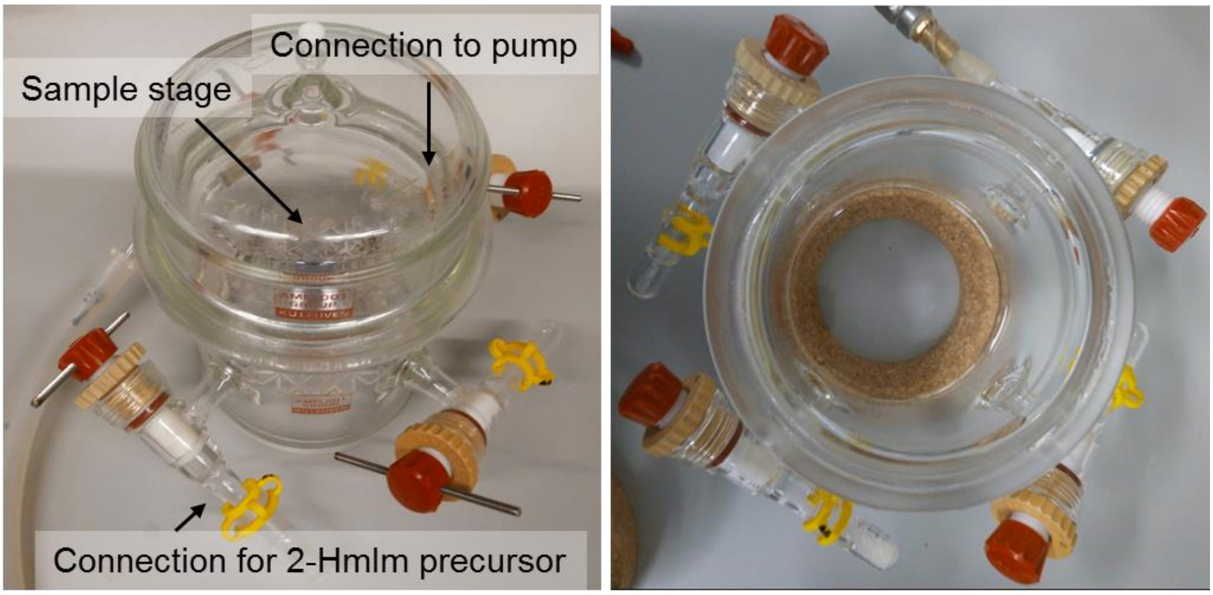

Supplementary Figure 1. Glassware reactor used for the conversion of metal oxide films to MOFs. During the conversion process, the glass reactor was evacuated and then placed in a pre-heated furnace.

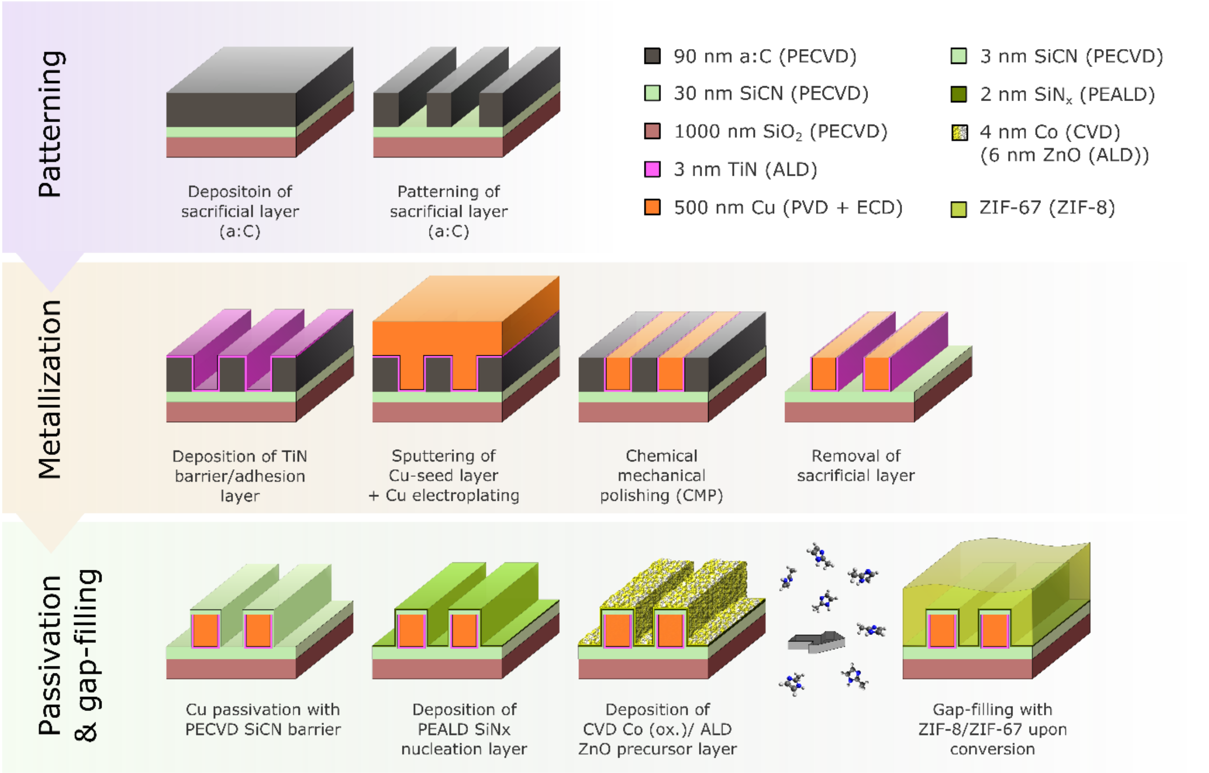

Supplementary Figure 2. The schematic process flow depicts the preparation of 45nm half-pitch fork-fork capacitors and the subsequent MOF-CVD gap-filling step. The following deposition methods were employed: ALD = atomic layer deposition, PECVD = plasma-enhanced chemical vapor deposition, PVD = physical vapor deposition (magnetron sputtering), PEALD = plasma-enhanced atomic layer deposition, ECD = electrochemical deposition (electroplating).

Supplementary Table 1. Comparison of relative atomic concentrations in vapor-phase deposited ZIF-8 and ZIF-67 films measured by XPS with those in ideal ZIF-8/ZIF-67 material determined from the unit cell composition.

Material	C 1s	N 1s	O 1s	Co 2p	Zn 2p
ZIF-67 (film)[a)]	61.9%	27.4%	3.9%	6.8%	-
ZIF-67 (ideal)	61.5%	30.7%	-	7.7%	-
ZIF-8 (film)[a)]	62.3%	27.2%	2.4%	-	8.2%
ZIF-8 (ideal)	61.5%	30.7%	-	-	7.7%

[a)] The XPS atomic concentrations are presented in angle integrated mode (all angles summed).

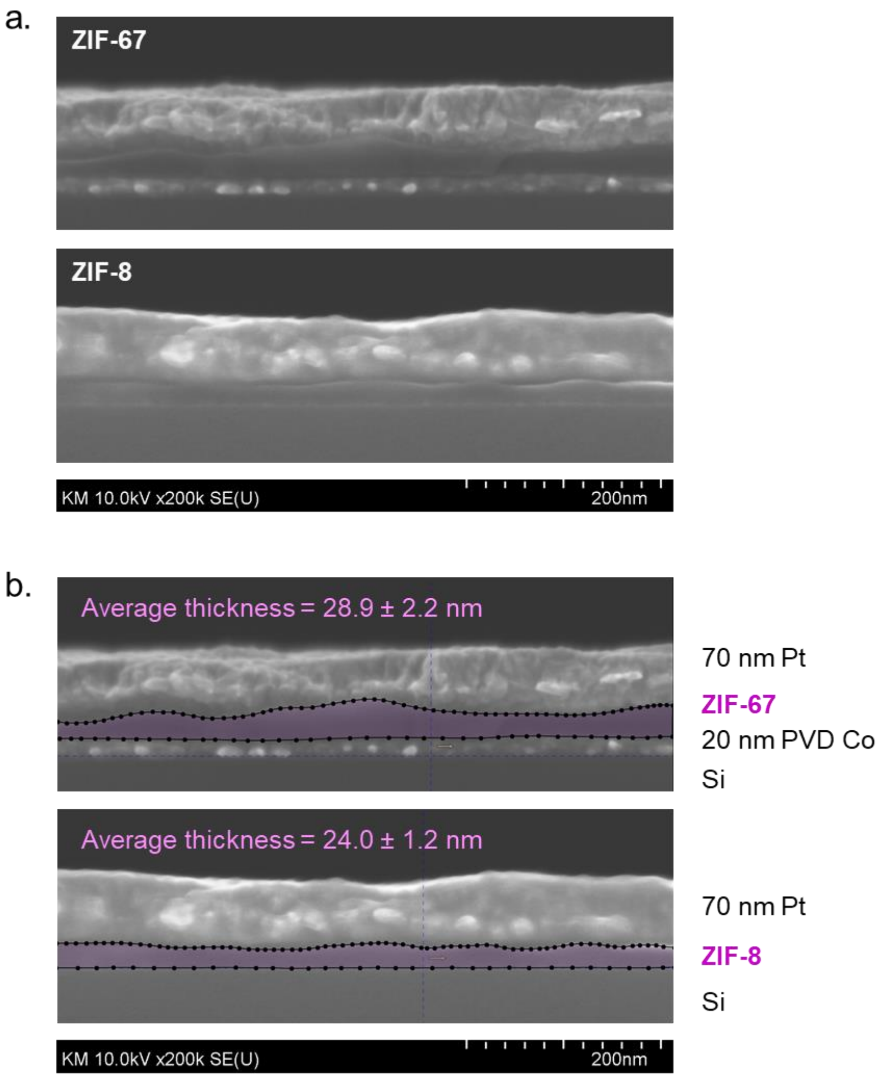

Supplementary Figure 3. Cross-sectional SEM images of vapor-phase deposited ZIF-8 and ZIF-67 films covered with 70 nm e-beam evaporated Pt. The thicknesses of the MOF coatings extracted from these images were used for calculation of k-values and for estimation of unconverted metal oxide thicknesses. (a) Original cross-sectional SEM images; (b) SEM images containing a contour used for calculation of the MOF layer thickness.

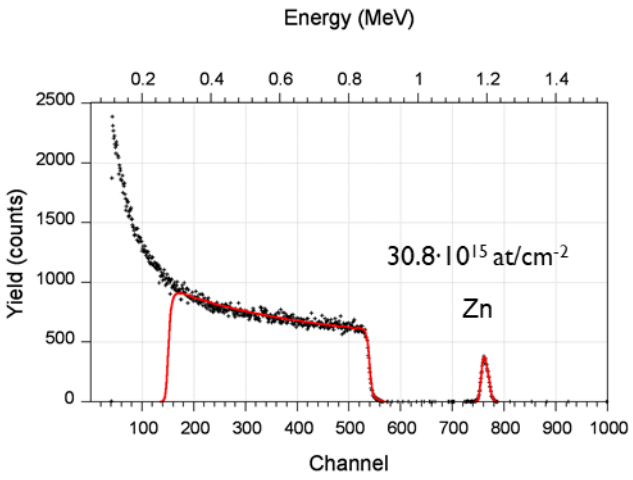

Supplementary Figure 4. RBS spectrum (black) and fitting curve (red) recorded on 8.9 nm thick ALD ZnO film deposited on top of a Si/SiO_2 substrate.

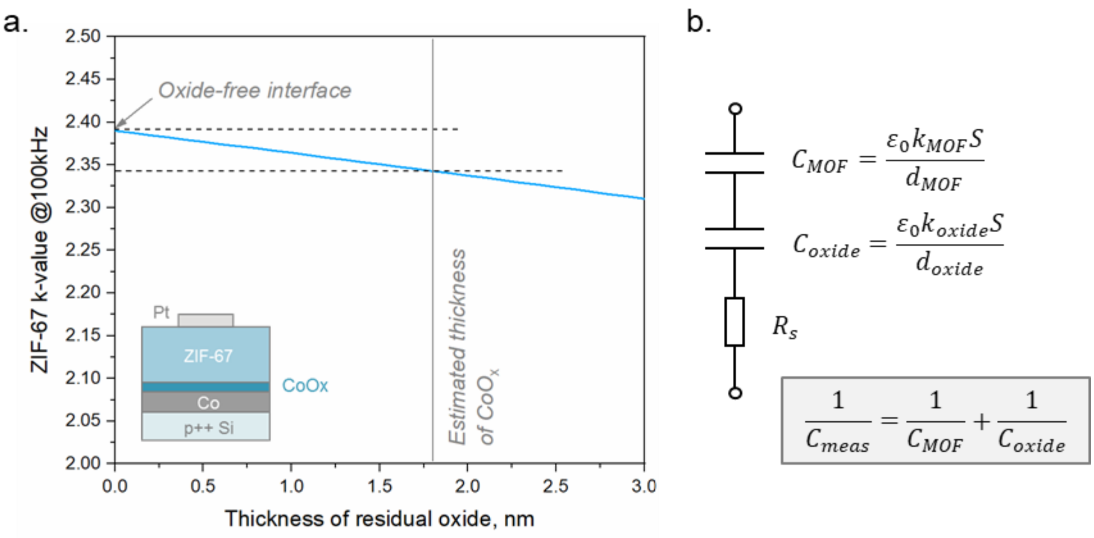

Supplementary Figure 5. Impact of a residual CoO_x layer. (a) Dependence of the extracted ZIF-67 k-value on the thickness of the interfacial CoO_x layer; (b) equivalent circuit scheme for impedance measurements on the MIM stack. During the calculation, k-value of the CoO_x residue layer was assumed to be equal to that of CoO[1].

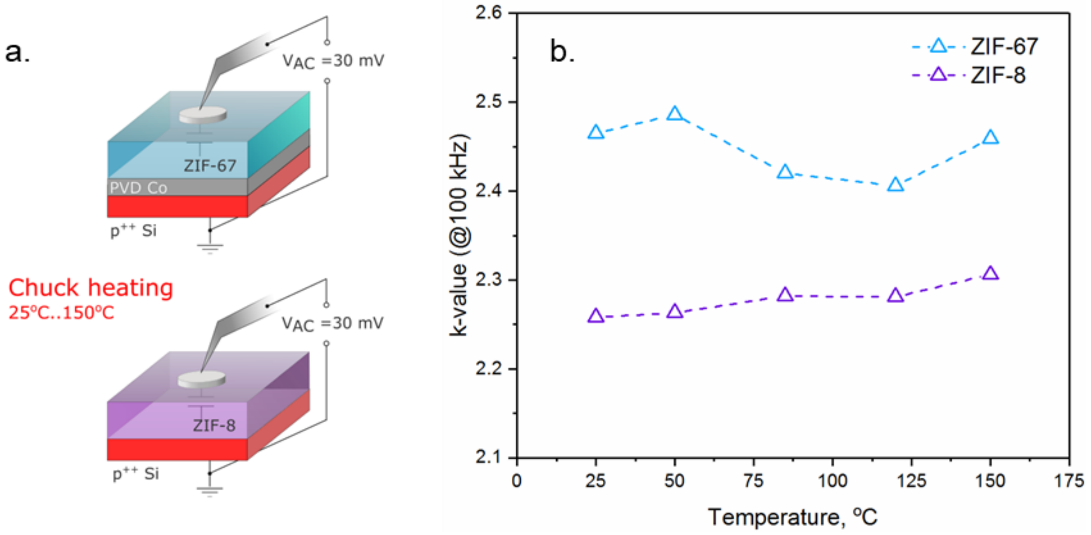

Supplementary Figure 6. Temperature dependence of ZIF-8 and ZIF-67 k-value measured on parallel-plate metal-insulator-metal capacitors with 0.03 mm^2 Pt top electrodes. (a) Schematic representation of the experiment; (b) k-values of ZIF films extracted from capacitance recorded on the chuck-heated devices in air.

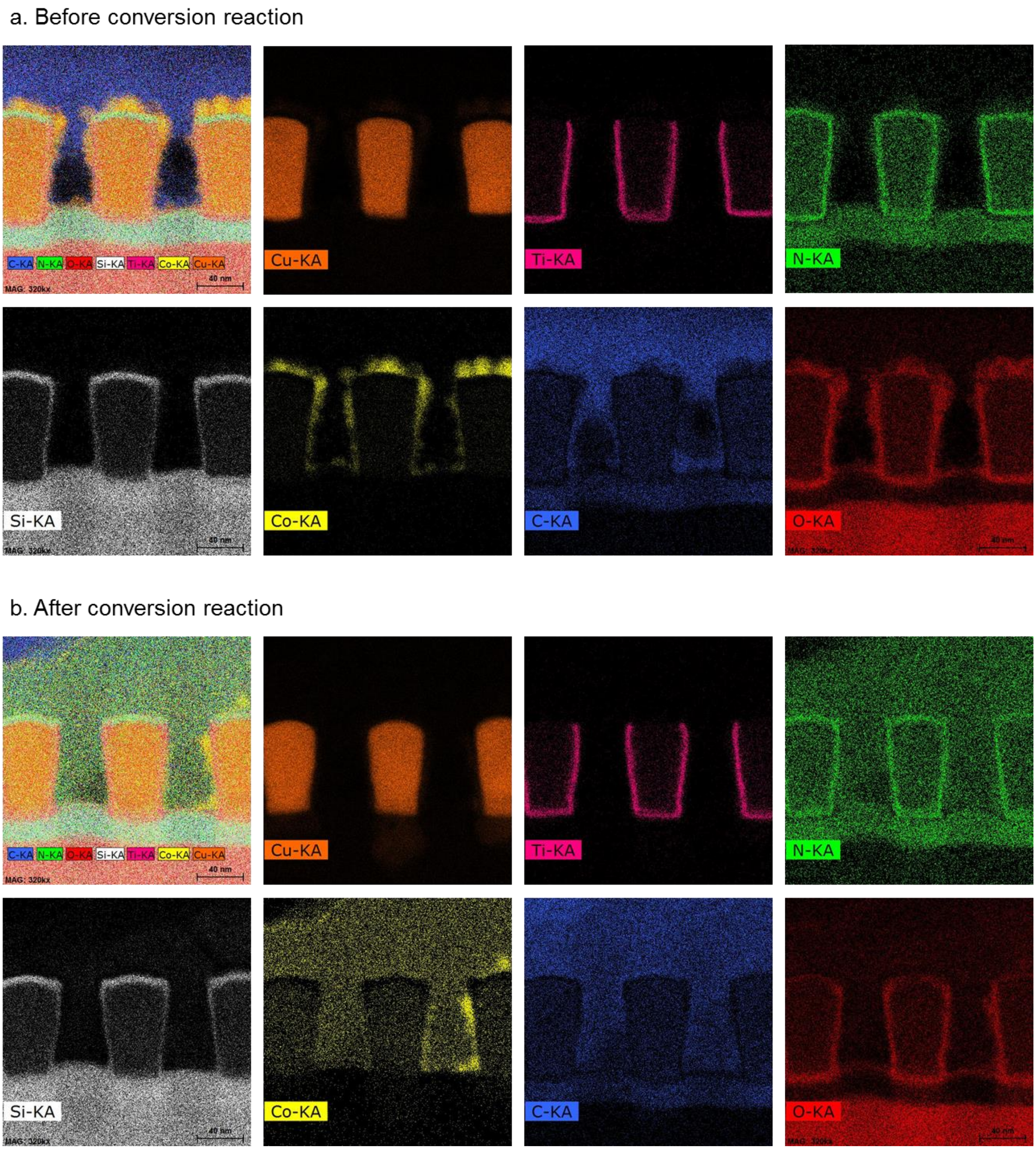

Supplementary Figure 7. EDS-TEM element maps recorded on the cross-section of 45 nm half-pitch fork-fork capacitor composed of passivated copper lines covered with oxidized CVD Co layer. (a) Before and (b) after the MOF-CVD conversion process.

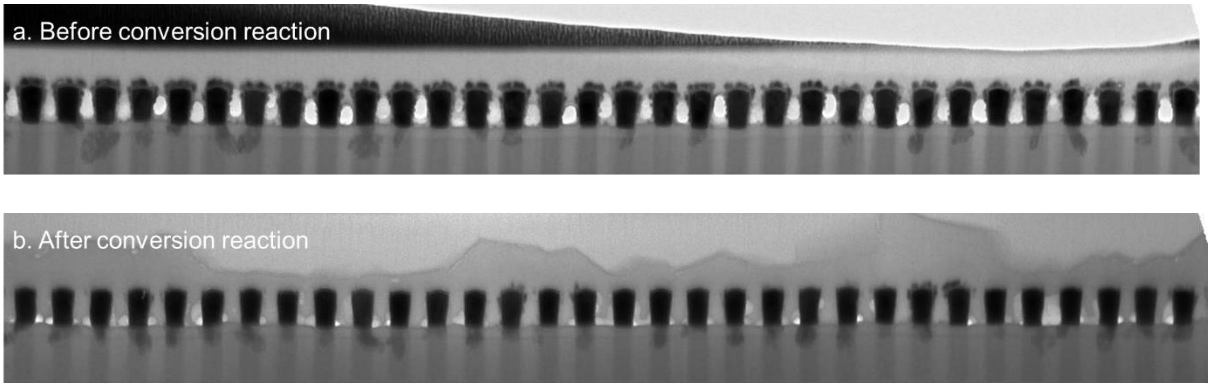

Supplementary Figure 8. TEM review images demonstrating the gap-filling performance of ZIF-67 phase on 45 nm half-pitch fork-fork capacitor. (a) Before and (b) after the conversion of oxidized CVD Co into ZIF-67 in vapors of 2-methylimidazole.

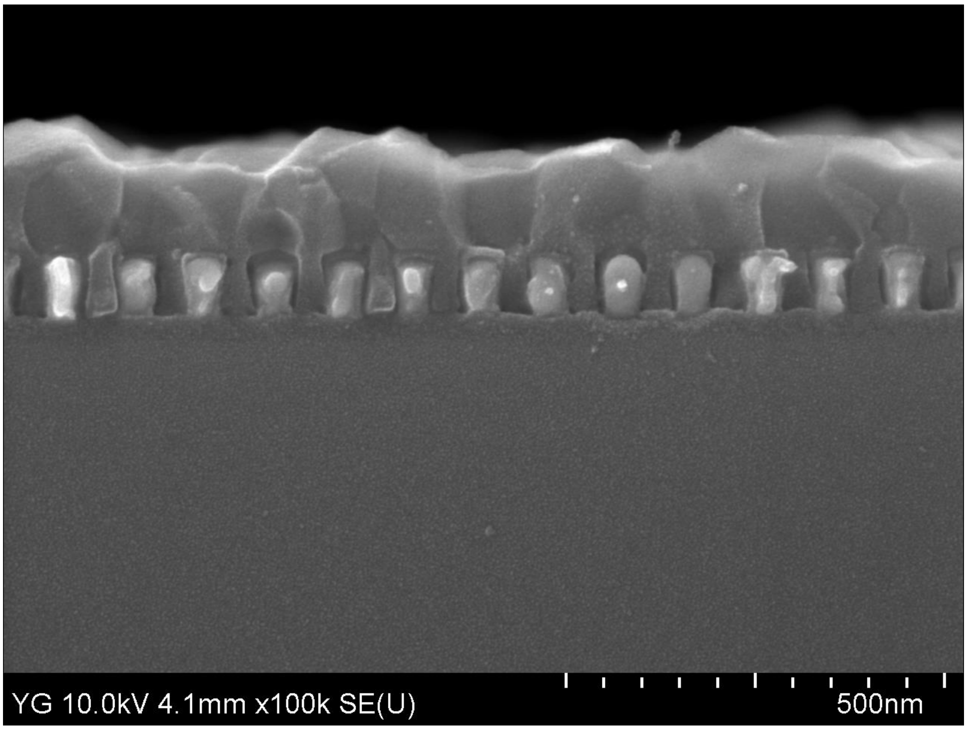

Supplementary Figure 9. Cross-sectional SEM image of manually cleaved substrate with 45 nm half-pitch fork-fork capacitors.

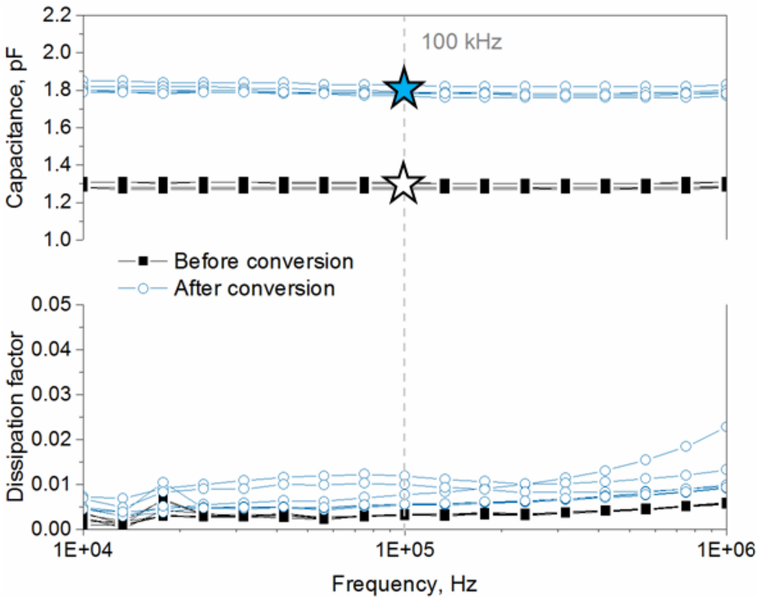

Supplementary Figure 10. Capacitance and dissipation factor measured on six 45nm half-pitch fork-fork capacitors in the frequency range 10 kHz-1MHz before and after MOF-CVD process. The average capacitance at 100 kHz measured on the samples after MOF-CVD reaction was used for extraction of effective k-value of the gap-filling phase.

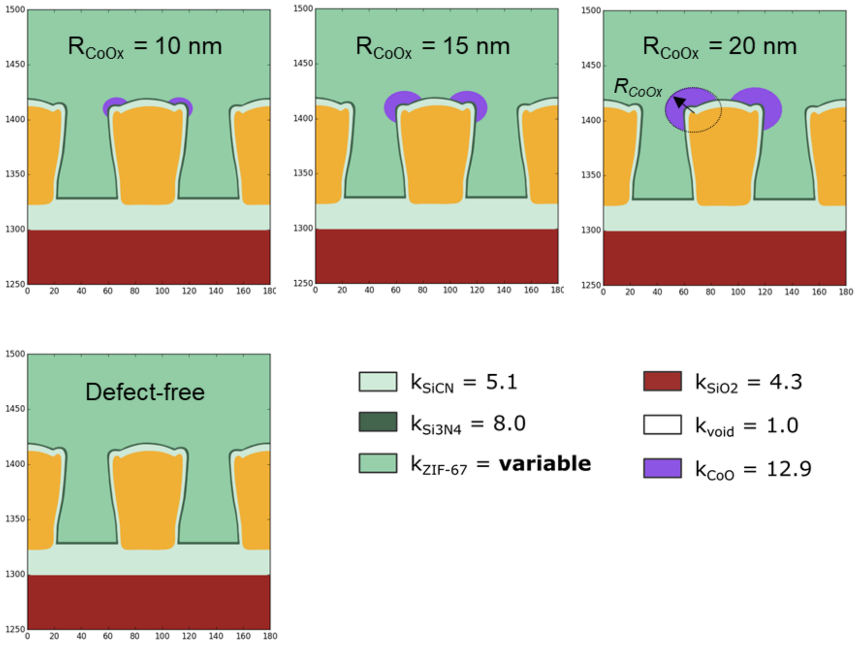

Supplementary Figure 11. Modelled cross-sections of 45 nm half-pitch fork-fork capacitors featuring CoO_x residues of different size (defect radius R_{CoOx}).

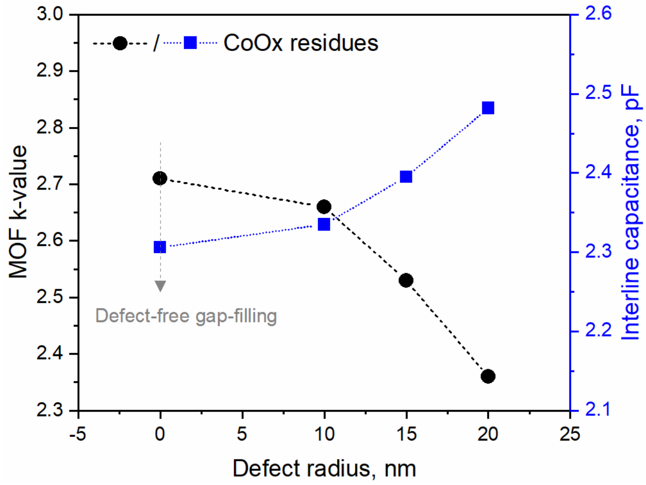

Supplementary Figure 12. The effect of CoO_x residues of different size on k-value of MOF-CVD ZIF-67 and on the total interline capacitance of a fork-fork device estimated by 2D capacitance simulations.

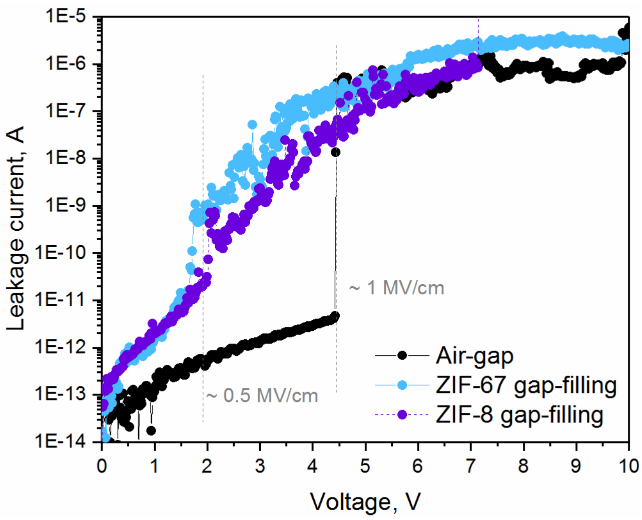

Supplementary Figure 13. Leakage current measured on 45 nm fork-fork capacitors with different gap-filling media. The ZIF-67 and ZIF-8 phases were formed via vapor phase conversion of oxidized CVD Co and ALD ZnO films, respectively.

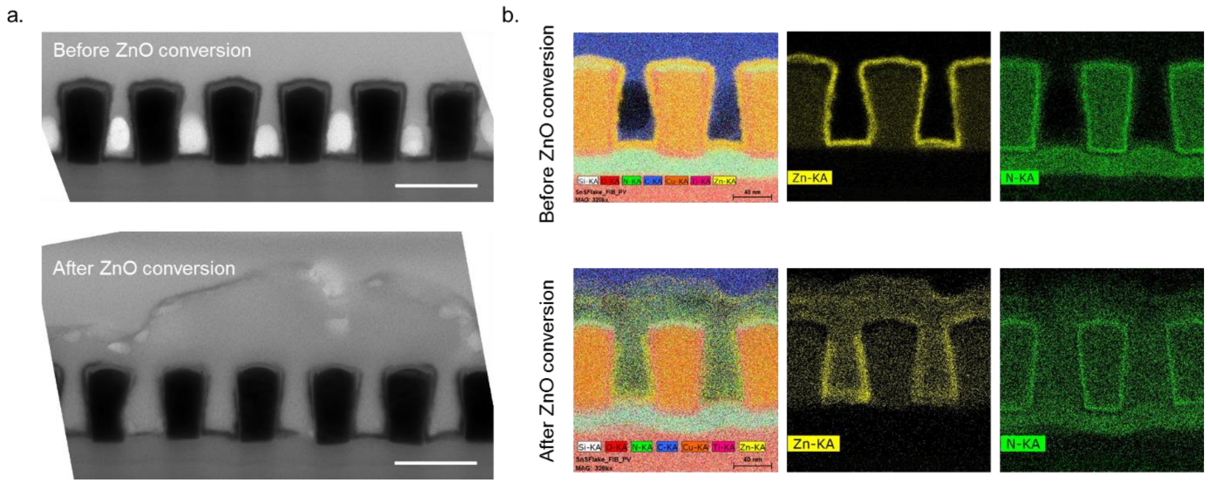

Supplementary Figure 14. TEM analysis of lamellas cut out from the capacitor device covered with 6 nm ALD ZnO before and after MOF-CVD process. (a) Bright-field TEM images; (b) EDS elemental maps. ALD ZnO is conformally covering the passivated copper lines. The 45 nm wide trenches appear gap-filled with ZIF-8 phase. There is also an evidence of a thin unconverted ZnO underlayer largely present inside the trenches.

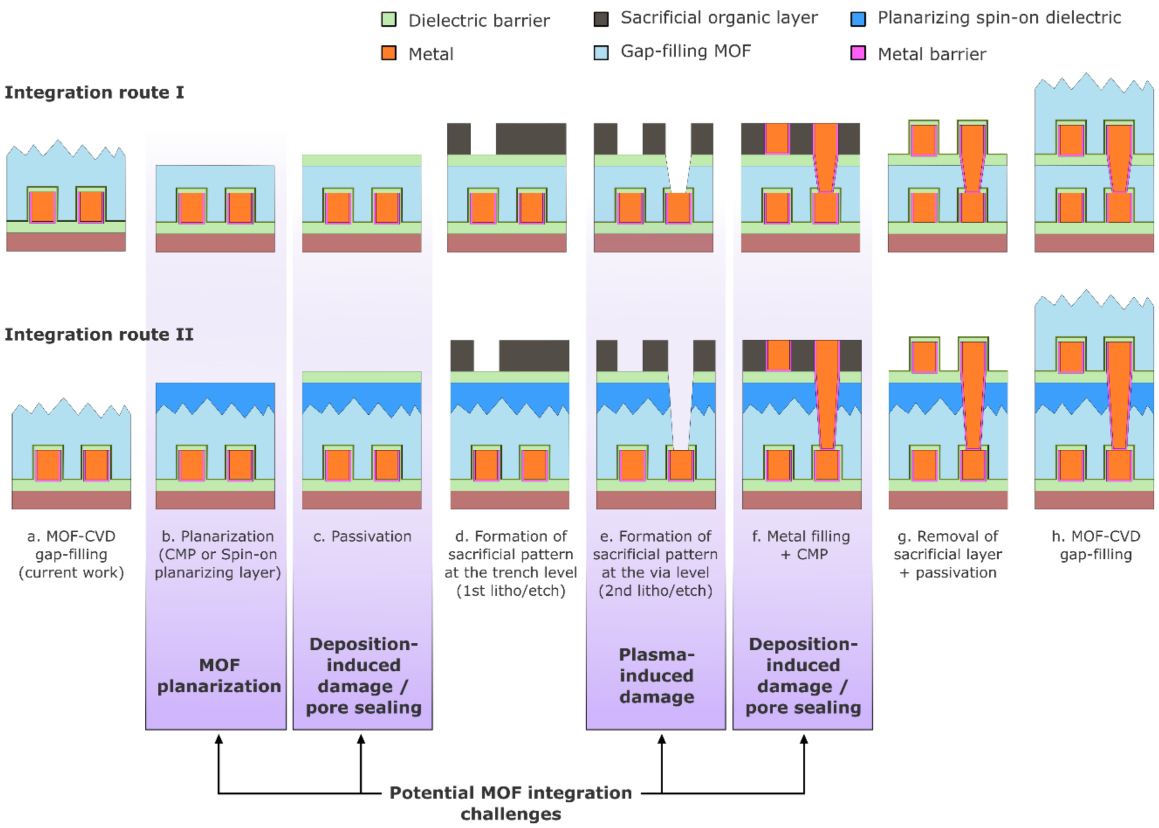

Supplementary Figure 15. Two metallization routes for the integration of gap-filling MOF-CVD dielectrics. The integration routes differ in the selected planarization approach (step b). In integration route I, chemical-mechanical polishing (CMP) is used to remove the film roughness. Integration route II uses a spin-on dielectric as a planarization coating. This extra dielectric could be a low-k variation of spin-on glass (SOG) material, which is routinely used in photolithography for local planarization before resist deposition. Besides planarization (step b), there are potential compatibility concerns at steps c and f related to deposition-induced damage and infiltration of subsequent layers (or building blocks thereof) into the porous MOF layer. Another question that needs to be addressed is the extent of plasma-induced damage during dry etching of the MOF layer at step e.

Supplementary references

1. Rao, K. V. & Smakula, A. Dielectric Properties of Cobalt Oxide, Nickel Oxide, and Their Mixed Crystals. *J. Appl. Phys.* **36**, 2031–2038 (1965).
